# External Validation of an AI mHealth Tool for Gingivitis Detection among Older Adults at Daycare Centers: A Pilot Study

**DOI:** 10.1016/j.identj.2025.01.008

**Published:** 2025-01-26

**Authors:** Reinhard Chun Wang Chau, Andrew Chi Chung Cheng, Kaijing Mao, Khaing Myat Thu, Zhaoting Ling, In Meei Tew, Tien Hsin Chang, Hong Jin Tan, Colman McGrath, Wai-Lun Lo, Richard Tai-Chiu Hsung, Walter Yu Hang Lam

**Affiliations:** a Faculty of Dentistry, The University of Hong Kong, Hong Kong Special Administrative Region, China; b Musketeers Foundation Institute of Data Science, The University of Hong Kong, Hong Kong Special Administrative Region, China; c Department of Computer Science, Hong Kong Chu Hai College, Hong Kong Special Administrative Region, China; d Faculty of Dentistry, The National University of Malaysia, Kuala Lumpur, Malaysia; e School of Dental Medicine, University of Pennsylvania, Philadelphia, Pennsylvania, US; f Eastman Dental Institute, University College London, London, UK

**Keywords:** Community dentistry, Gingivitis, Machine learning, Mobile health, Periodontal diseases, Telemedicine

## Abstract

**Objectives:**

Periodontal disease is a significant public health concern among older adults due to its relationship with tooth loss and systemic health disease. However, there are numerous barriers that prevent older adults from receiving routine dental care, highlighting the need for innovative screening tools at the community level. This pilot study aimed first, to evaluate the accuracy of GumAI, a new mHealth tool that uses AI and smartphones to detect gingivitis, and the user acceptance of personalized oral hygiene instructions provided through the new tool, among older adults in day-care community centers.

**Methods:**

Participants were invited from 3 day-care community centers. Intraoral photographs were captured and assessed by both GumAI (test) and a panel consisting of 2 calibrated periodontists and a dentist (benchmark). Mean sensitivity, specificity, positive predictive value (PPV), negative predictive value (NPV), accuracy, and F1 score were calculated to determine GumAI's diagnostic performance in comparison to the benchmark. User acceptance with this tool was assessed using 2 Rasch Theory-based 5-point Likert-type questions.

**Results:**

44 participants were recruited out of 80 invited older adults. GumAI demonstrated a sensitivity of 0.93 and specificity of 0.50 compared to the panel‘s assessments, with a PPV of 0.90 and NPV of 0.56. The accuracy and F1 scores were 0.85 and 0.91, respectively. All participants expressed high acceptance of the process.

**Conclusion:**

GumAI demonstrates high sensitivity, PPV, accuracy, and F1 score compared to the panel's assessments but falls relatively short in specificity and NPV. Despite this, the tool was highly accepted by older adults, indicating its potential to enhance gingivitis detection and oral hygiene management in community settings. Further refinements are necessary to improve specificity and validate usability measures.

**Clinical Relevance:**

This study may pave the way for broader applications of mHealth systems in community settings, enabling greater health coverage and addressing oral health disparities.

## Introduction

Periodontal disease is a leading cause of tooth loss and has been associated with various systemic health issues, including cardiovascular disease, diabetes, and respiratory infections.^1^ This bidirectional relationship between periodontal disease and general health highlights the importance of maintaining good oral hygiene and early detection of gingivitis, particularly among older adults with an increased risk of developing related health complications.^2^^,^^3^ Gingivitis, an early stage of periodontal disease characterized by inflammation and bleeding of the gingival tissues surrounding the teeth, is one of the most prevalent forms of periodontal disease globally.^4^^,^^5^ It serves as a key indicator of periodontal inflammation and can progress to periodontitis if left untreated.^6^^,^^7^

With an aging population worldwide, the prevalence of periodontal disease among older adults has become a growing public health concern, as oral health plays a vital role in overall well-being and quality of life.^2^^,^^8^ Factors such as relative lack of oral health awareness and financial constraints can be barriers to receiving routine dental care, which typically involves frequent dental visits and examinations. This is particularly relevant for older adults, especially those living in communities.^9^, ^10^, ^11^, ^12^ Community settings, defined as geographically defined areas where residents (older adults) linked by shared social ties reside,^13^ present unique challenges in providing routine dental care.^14^ Consequently, older adults in these settings may face common barriers to accessing periodontal healthcare, exacerbating the risk of unmanaged gingivitis and its progression.

Rapid advancements in artificial intelligence (AI) and smartphone technology offer promising solutions for mhealth to address the challenges faced by older adults.^15^ AI, with its ability to perform tasks typically requiring human intelligence,^16^^,^^17^ has shown significant potential in dentistry, including detecting and managing periodontal diseases,^18^^,^^19^ carries,^20^ sinusitis,^21^, and tooth loss.^22^^,^^23^ These advancements coincide with the increasing accessibility of smartphones, with estimates suggesting that over 80% of the global population owned a smartphone by the end of 2023.^24^ By harnessing the power of AI and readily available smartphone technology, it is possible to create practical tools that empower individuals with accurate diagnostic capabilities for detecting and preventing gingivitis.^18^ Such tools are particularly effective when used for oral hygiene maintenance within a 6-month period and can help bridge the gap in accessible dental care for older adults in community settings.^25^^,^^26^

This pilot study aimed to evaluate the accuracy of GumAI, a mobile health (mHealth) tool that uses artificial intelligence (AI) and smartphones to detect gingivitis,^18^ as well as user acceptance with personalized oral hygiene instructions provided through this mHealth tool among older adults in day-care community centers. While GumAI was reported to have a high sensitivity of 0.92 and specificity of 0.94 in a controlled clinical setting,^18^ it remained unknown whether it would perform satisfactorily in community settings where contextual factors might differ significantly. This study aimed to evaluate whether GumAI can meet the principles for high-quality, high-value testing (the sum of sensitivity and specificity ≥1.50)^27^ in detecting gingivitis among older adults in community settings compared to evaluations conducted by dental professionals. Additionally, the study sought to assess user acceptance of the mHealth tool in real-world community environments.

## Methods

**Fig. 1:**
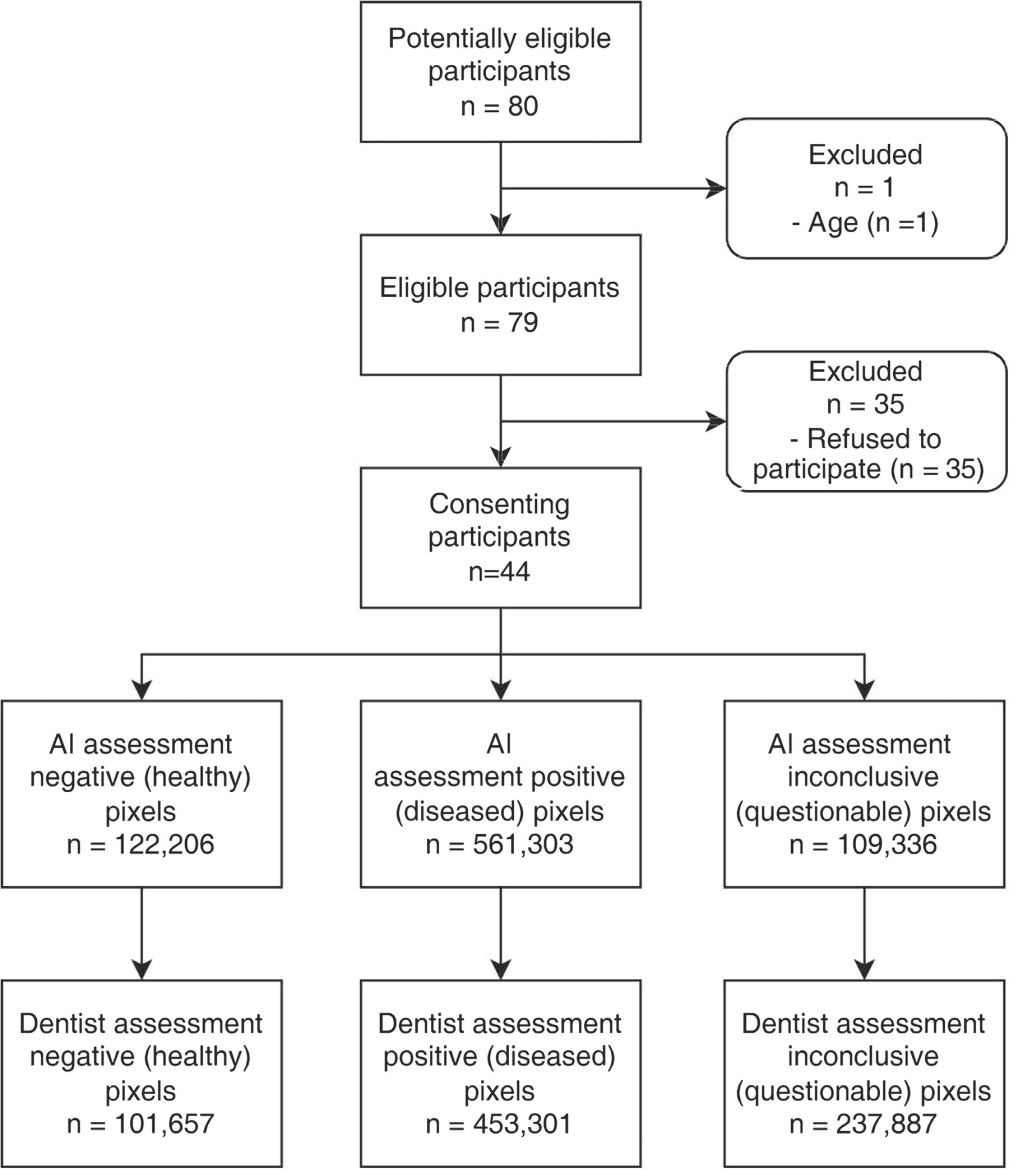
The STARD-2015 flow diagram of this study.

This study was approved by the Institutional Review Board of the University of Hong Kong/Hospital Authority Hong Kong West Cluster (HKU/HA HKW IRB), Hong Kong Special Administrative Region, China (Reference Number: UW 21-447), and the Research, Ethics/Safety Sub-Committee (RESS) of Hong Kong Chu Hai College, Hong Kong Special Administrative Region, China (Reference Number: RESS/2022/06/006). This study was registered on ClinicalTrials.gov (Reference Number: NCT05685355) and was conducted in accordance with the Standards for Reporting Diagnostic Accuracy (STARD) 2015 statement (Figure 1).^28^

### Study design

A comparative diagnostic accuracy design was employed by this pilot study to evaluate the performance of GumAI, a mobile health (mHealth) gingivitis screening tool, against professional visual assessments conducted by a panel of periodontists and dentists. The study was conducted prospectively in Cantonese at 3 day-care community centers that serve as hubs for older adults’ daily activities.

### Participants

Participants were recruited from older adults attending a community dental outreach program at 3 local community centers between February 8, 2023, and March 24, 2023. These older adults were registered members of the community centers and were invited by the staff of the respective community centers based on their membership records. The community center staff were briefed about a free AI-powered dental screening initiative, but no scripts were provided. Eligibility was assessed by an assessor (RCWC). All participants were informed about the nature of this pilot study, which aimed to evaluate the performance of a mHealth gingivitis screening tool, and written consent was obtained from all eligible and consenting participants by the same assessor. As compensation, each participant received 1 toothpaste after completing the study. Quota sampling was applied, and based on the calculated sample size, recruitment continued until there were sufficient participants with healthy gingiva (healthy condition) and with gingivitis (diseased condition).

The inclusion criteria were:

- Subjects who are aged 60 years or above.
- Subjects who have the ability to use community center facilities.
- Subjects who have the capability to communicate and provide informed consent in Cantonese/Chinese.

The exclusion criteria were:

- Subjects who received periodontal treatments within the last 3 months.

### Instruments

**Fig. 2:**
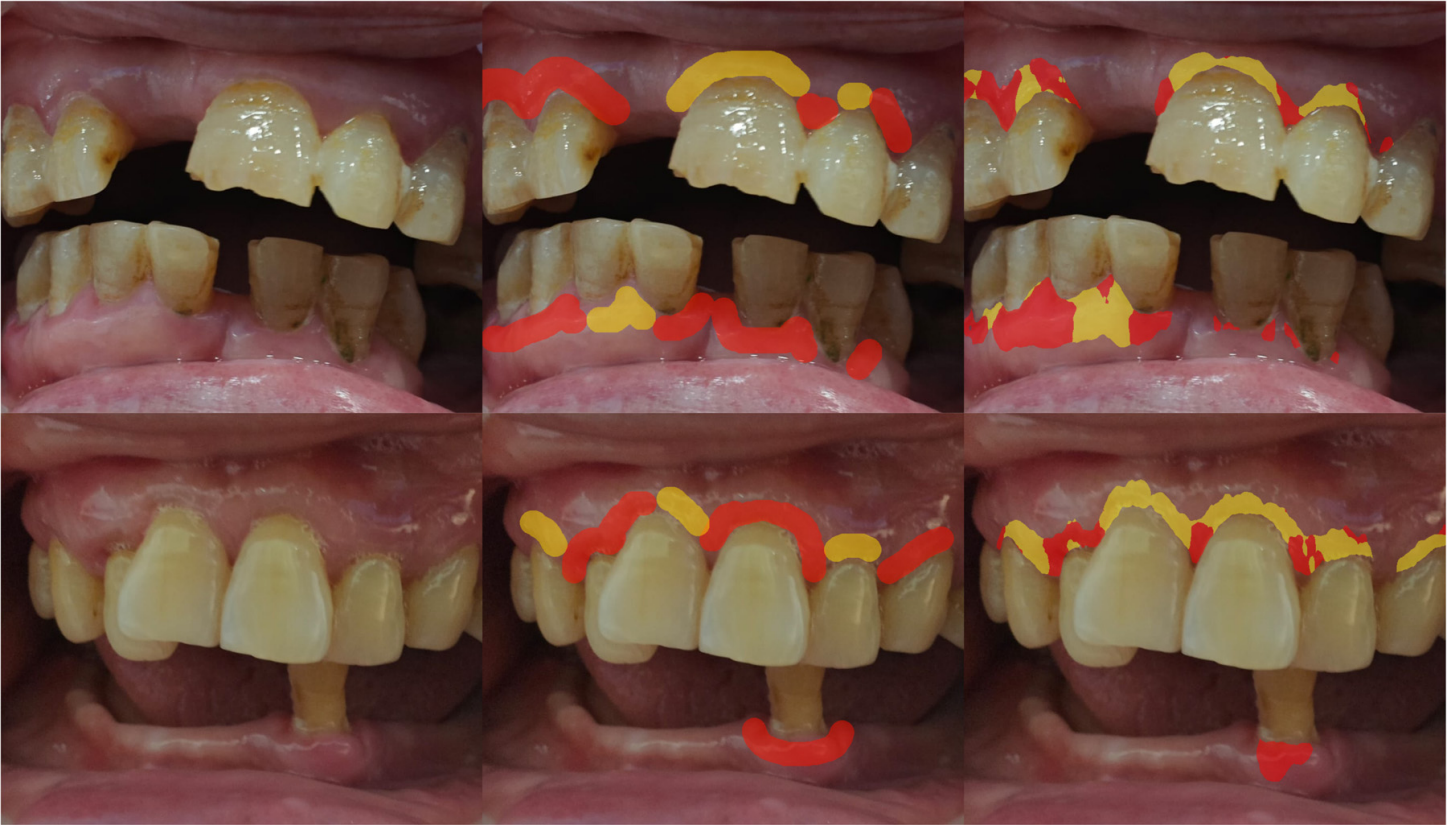
Examples of Gum detection results compared to those of the calibrated periodontist panel. Left column: Intraoral photographs of 2 older adults. Middle column: Gum condition diagnosed by the periodontist panel. Right column: Gum condition diagnosed by the AI screening tool. Green = *healthy*, red = *diseased*, and yellow = *questionable*.

GumAI is a mHealth screening tool designed to assess gingival health using intraoral photographs captured by a smartphone and analyzed through AI. Its primary function is to receive photographs from users and generate annotated images as screening results. The tool utilizes the DeepLabv3+ machine learning architecture and operates on a Linux operating system (Ubuntu 22.04, Canonical) powered by 4 Graphic Processing Units (GPUs) (RTX 4090, Nvidia). It was trained on 454 standardized intraoral photographs and previously demonstrated a sensitivity of 0.92 and specificity of 0.94 in a controlled clinical setting.^18^ For each uploaded photograph, GumAI assesses the gingival health condition of individual sites on a pixel level 3mm apical to the margin, from the left first premolar to the right first premolar on both maxillary and mandibular arches (FDI tooth numbers 14-24 and 34-44), classifying them as healthy (green), questionable (yellow), or diseased (red) (Figure 2). This constituted the test group in this pilot study.

The visual assessments of intraoral photographs served as the benchmark group in this study and were independently conducted by 2 calibrated periodontists (THC and HJT) who were blinded to the AI assessment results without changing the resolutions of the photographs. The assessments were performed with a 3mm brush along the gingival margin. For calibration, both periodontists were provided with 20 pre-labeled images and assessment guidelines. They each independently assessed an additional set of 20 images, which were then reviewed for accuracy. Any disagreement was addressed through feedback to resolve conflicts. Both periodontists use high-resolution screens,^29^ with a 2560 × 1600 and a 2388 × 1668 display, respectively. Any disagreement between the 2 periodontists was resolved through discussions with a calibrated dentist (ZTL).

The assessments of GumAI and the periodontists categorized gingival health using the same 3 classifications: healthy, questionable, and diseased. These classifications were adapted from the “Gums and tissues” category of the Oral Health Assessment Tools (OHATs) to label the areas of interest as follows:^30^, ^31^, ^32^

- Healthy: pink, smooth, no bleeding
- Questionable: red, rough, swollen
- Diseased: white/red patches, generalized redness, ulcers, swollen, bleeding

The user acceptance of the mHealth system and the personalized oral hygiene instructions provided through this mHealth tool were rated by the participants using 2 items adopted from a 14-item Rasch Theory Questionnaire for Assessing User Satisfaction With Mobile Health Apps:^33^

1. "This (mHealth) tool has helped me to assess my gum health."
2. "I am satisfied with the personalized oral hygiene instructions provided through this (mHealth) tool."

Each response was logged on a 5-point Likert scale, with 1 indicating “strongly disagree” and 5 indicating “strongly agree."

As there was no validated Cantonese version of the questionnaire available, these questions were translated into Cantonese by a native Cantonese speaker (RCWC) and reviewed by a bilingual colleague (WYHL) to ensure clarity and cultural appropriateness.

### Data collection

Frontal-view intraoral photographs were captured using the rear-facing primary camera (50MP Sony IMX890, Sony) of a smartphone (OnePlus 11, OnePlus) by an operator who stood in front of each participant's face, with the smartphone aligned parallel to the participants’ frontal plane. The photographs were captured under the resolution of 1600 × 1200 in High Dynamic Range (HDR) mode to ensure a clear view of the incisors, canines, and first premolars without additional lighting. The remaining camera settings were maintained at their default values. The intraoral photographs in this study were not strictly standardized, as they would be in controlled clinical settings, to assess the use of the mHealth system on a smartphone without any specialized instrument in real-life community settings.^34^

Subsequently, the captured intraoral photographs were uploaded to GumAI for analysis by the operator. These photographs were also visually assessed by 2 calibrated periodontists to evaluate the participants’ gingival health, serving as a benchmark for external validation.

**Fig. 3:**
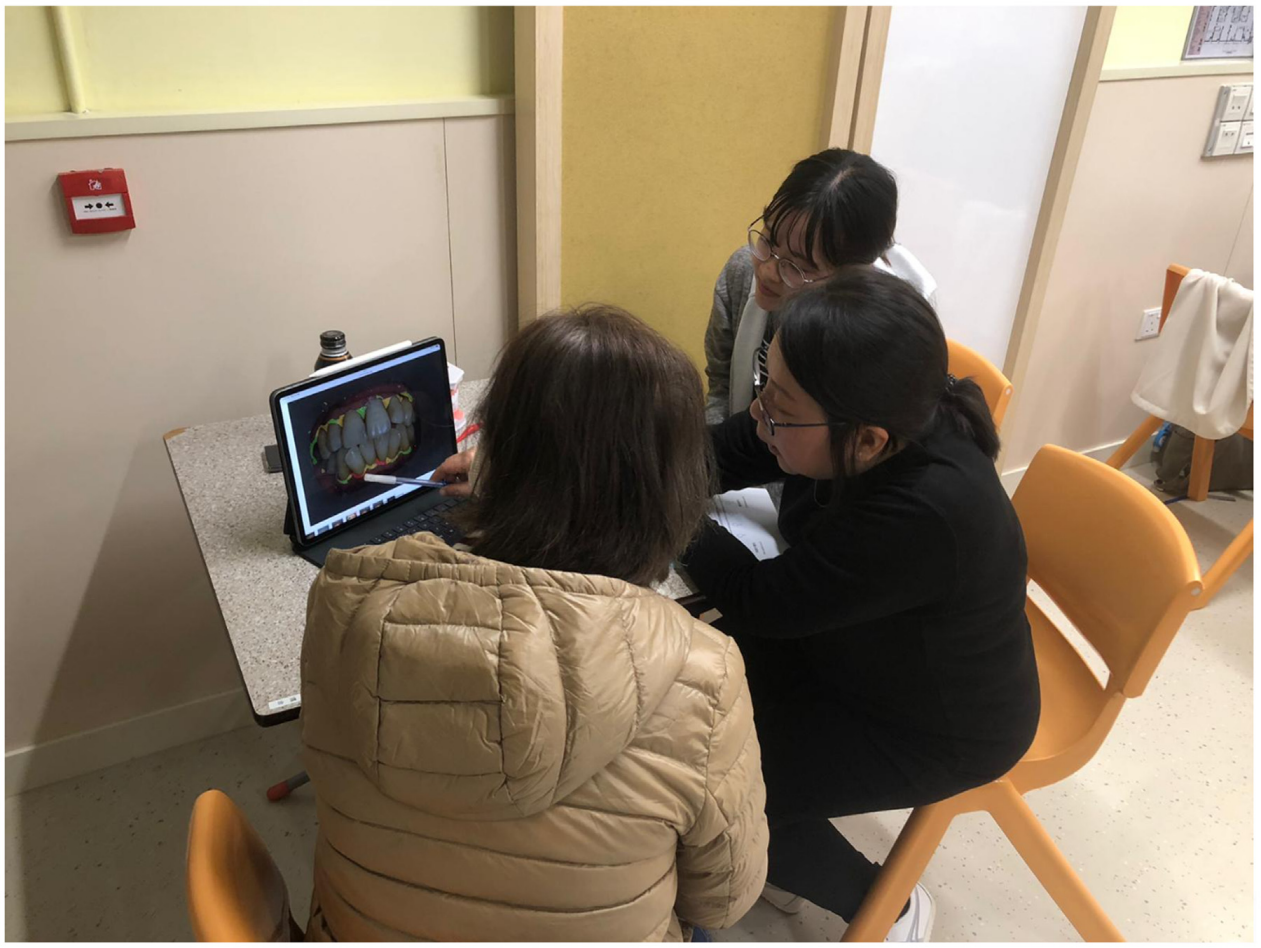
An illustration of the process of providing personalized oral hygiene instructions to an older adult based on the result of GumAI.

Finally, a dental surgery assistant (DSA) with over 20 years of clinical experience provided personalized oral hygiene instructions to each participant based on their GumAI photographs (Figure 3). For gingival sites labeled as diseased (red) or questionable (yellow), the DSA offered additional guidance on proper tooth brushing and interdental cleaning.^35^ Each participant was provided with an electronic copy of the AI screening results upon request to facilitate proper tooth brushing and interdental cleaning at home. At the conclusion of the session, the participants independently completed the paper-based user acceptance questionnaire, with helpers reading out the questions and answer options if needed.

### Data preparation

The assessments conducted by GumAI were compared against the visual assessments made by the panel of calibrated periodontists, which served as the benchmark and external validation reference. The panel was blinded to GumAI's assessments to minimize any potential bias.

### Measurements

The accuracy of GumAI in determining the healthy and diseased gingival sites computed by images and was evaluated using the mean values of the following statistical measures:^36^

- Sensitivity: Proportion of true positive gingivitis (diseased) sites correctly identified by GumAI.
- Specificity: Proportion of true negative (healthy) sites correctly identified by GumAI.
- Positive Predictive Value (PPV): Probability that sites identified as diseased by GumAI are truly diseased according to the benchmark.
- Negative Predictive Value (NPV): Probability that sites identified as healthy by GumAI are truly healthy according to the benchmark.
- Accuracy: The proportion of sites correctly identified by GumAI as either having gingivitis (diseased) or being healthy, out of all the sites examined.
- F1 Score: The harmonic means of GumAI's precision (proportion of sites correctly identified as having gingivitis out of all sites flagged by GumAI as diseased) and recall (sensitivity - the proportion of sites with gingivitis correctly identified by GumAI).

The calculations were based on the following formula:

Sensitivity=TP/(TP+FN),

Specificity=TN/(TN+FP),

PPV=TP/(TP+FP),

NPV=TN/(TN+FN),

Accuracy=(TP+TN)/(TP+TN+FP+FN),

F1Score=2×{[TP/(TP+FP)]×[TP/(TP+FN)]}/{[TP/(TP+FP)]+[TP/(TP+FN)]},

where “true-positive”(TP) indicates the AI tool's correct detection of a diseased status, while “true-negative”(TN) indicates its correct detection of a healthy status. On the other hand, “false-positive”(FP) and “false-negative”(FN) represent instances where the AI tool misclassified healthy sites as diseased and diseased sites as healthy, respectively. The “questionable” classification is not included in the calculation as it includes sites that the assessors cannot determine as “healthy” or “diseased” and is therefore not considered according to the STARD guidelines.^28^

### Sample size determination

A sample size of 12 participants for each healthy and diseased condition, totaling 24 participants, was set for this pilot study to evaluate the accuracy of GumAI in determining the healthy and diseased gingival sites. This aligns with the recommended minimum sample size for pilot studies suggested,^37^ and allows for initial assessment of feasibility, study procedures (e.g., recruitment, data collection), and user acceptance, which will inform the design of a subsequent, larger-scale study.

### Statistical analysis

Data were analyzed using a statistical Python library (Scikit-learn version 1.5.2.).^38^ Descriptive statistics summarized participant sociodemographics and questionnaire responses. Sensitivity, specificity, PPV, NPV, accuracy, and F1 score were calculated to assess the diagnostic performance of GumAI in determining the healthy and diseased gingival sites relative to the benchmark assessed by the calibrated periodontist panel.^36^

## Results

In this study, 80 older adults attended the 3 day-care community centers, ranging in age from 54 to 91. Out of the 80 older adults, 28 (35.0%) were from Sham Shui Po District, 30 (37.5%) were from Tsuen Wan District, and 22 (27.5%) were from Kwai Tsing District. Among these participants, 72.5% were female (n = 58) and 27.5% were male (n = 22), with a mean age of 76 and a standard deviation of 7.4. Of the 80 subjects, 44 (55.0%) were recruited for the study. One participant (1.3%) was deemed ineligible due to being below the age of 60, while the remaining 35 (43.8%) individuals declined participation, citing concerns about being photographed.

A total of 44 frontal-view intraoral photographs were captured and analyzed. GumAI accurately identified 41764 healthy pixels and 382909 diseased pixels (Table 1), achieving a sensitivity of 0.93 and a specificity of 0.50 compared to the calibrated periodontist panel's assessments. The Cohen's kappa coefficient between the 2 periodontists was 0.95 (95% CI: 0.94, 0.96), indicating excellent inter-rater reliability.

**Table 1:** The Confusion matrix of GumAI in detecting gingivitis compared to the periodontist panel's assessment.

	Benchmark positive	Benchmark negative
Predicted positive	382909	41059
Predicted negative	31036	41764

The PPV and NPV compared to the calibrated periodontist panel's assessments were 0.90 and 0.56, respectively. The accuracy of GumAI compared to the calibrated periodontist panel was 0.85. The resulting F1 Score of the comparison between GumAI and the calibrated periodontist panel was 0.91, which was considered “very good” and indicated strong performance for the assessment tasks.

All 44 participants (100%) rated either "strongly agree" or "agree" under "This (mHealth) tool has helped me to assess my gum health." and "I am satisfied with the personalized oral hygiene instructions provided through this (mHealth) tool." (Table 2).

**Table 2:** Participant ratings of AI-powered oral hygiene instructions.

1. This (mHealth) tool has helped me to assess my gum health.	Percentage (%)
Strongly Agree	36.4% (16/44)
Agree	63.6% (28/44)
Neutral	0% (0/44)
Disagree	0% (0/44)
Strongly Disagree	0% (0/44)

2. I am satisfied with the personalized oral hygiene instructions provided through this (mHealth) tool.	
Strongly Agree	38.6% (17/44)
Agree	61.4% (27/44)
Neutral	0% (0/44)
Disagree	0% (0/44)
Strongly Disagree	0% (0/44)

## Discussion

The results of this external validation study provided preliminary data on the diagnostic performance and user acceptance of a mHealth tool, GumAI, among older adults in community settings. GumAI demonstrated high sensitivity (0.93) and moderate specificity (0.50) in detecting gingivitis compared to the assessments made by the calibrated periodontist panel.^39^ The diagnostic performance of this tool slightly fell below the threshold for high-quality, high-value tests, where the sum of sensitivity and specificity should be ≥1.50. Still, a sum of 1.43 was close to the high-value threshold and represented good potential in a pilot study.^40^^,^^41^ This shortfall was primarily due to the moderate specificity value yet the high sensitivity (0.93) and positive predictive value (PPV) (0.90) observed in this pilot study suggest that most cases of gingivitis within the community settings detected by GumAI were in agreement with the panel of periodontists, which is crucial for early disease detection and intervention. The high accuracy (0.85) and F1 Score (0.91) also indicate the promising assessment capabilities of GumAI.

These findings largely align with previous research investigating AI tools in dental diagnostics with varying accuracies. For instance, 7 studies reported accuracies ranging from 0.74 to 0.78 when using their AI tools to diagnose gingivitis from intraoral photographs and accuracies ranging from 0.68 to 0.74 when diagnosing from fluorescent intraoral images.^42^ However, all these studies were performed in a controlled clinical setting and required specialized instruments. In a prior study of GumAI, the sensitivity was 0.92, and the specificity was 0.94 in a controlled clinical setting.^18^ What sets this study apart is its examination of GumAI in a real-world community setting without any specialized instrument, demonstrating its applicability beyond controlled environments.

The specificity observed in this community-based study (0.50) is lower than that observed in the previous study (0.94). This decrease is expected since the measurement was conducted in a community setting where controlled standardizations are not feasible. This result suggests that contextual and procedural factors, such as background visual complexity and the method of capturing photographs, may influence the AI tool's diagnostic performance. While studies suggest minimal differences between the color accuracy of single-lens reflex (SLR) and smartphone cameras under controlled environments,^43^ community settings often feature cluttered and dynamic backgrounds. These conditions can introduce visual noise, such as varied lighting and light reflection from saliva during photograph captures, which can obscure relevant features that the AI relies on for accurate disease detection. Additionally, the diverse participants’ clinical and demographic characteristics in community settings—including oral conditions, biotypes, age and behavioral variations—can introduce a wide range of appearances that may impact the AI. These combined factors can result in an increase in false positives or negatives, thereby diminishing the overall specificity of the AI tool in real-world applications.

The 100% positive ratings reflect a high level of acceptance of GumAI among older adults in community settings. Participants found the personalized oral hygiene instruction provided through this mHealth tool helpful and easy to follow. The visual identification of diseased gingival sites enables older adults to perceive the exact location of diseased gum and aids in their self-management. The high level of acceptance is essential for the adoption and sustainability of mHealth tools in community settings, especially among populations facing barriers to accessing routine dental care.

The limitations of this pilot study must be acknowledged, including the small sample size of 44 participants, which may restrict the generalizability of the findings. While this study was conducted across 3 community centers in the city, the sample may not fully represent the broader older adult population. The lack of formal validation for the translated questions could affect their reliability and validity. Contextual differences between community and controlled clinical settings, like lighting variations, may influence the mHealth tool's diagnostic performance. The fact that 43.8% (n = 35) of older adults declined participation due to concerns about being photographed highlights the importance of promoting and explaining the use of photographs in disease management and addressing privacy and consent issues. To improve acceptance and engagement among older adults, future AI tool implementations should prioritize transparent data practices and implement robust measures to protect user privacy.

The increasing accessibility of smartphones, coupled with the high sensitivity of GumAI in gingivitis detection, highlights its potential as a valuable teledentistry tool for gingivitis screening in community settings,^44^^,^^45^ despite the relatively limited application of teledentistry by date.^46^ However, caution should be exercised as the moderate specificity may result in some gum diseases being overlooked. Positive feedback from participants further supports the feasibility and acceptability of the mHealth tool, suggesting that with improvements, it could help address gaps in access to dental care for older adults.

Future research should incorporate longitudinal studies with larger sample sizes and more diverse settings to better evaluate the impact of using mHealth tools on oral health outcomes and healthcare utilization among older adults. As AI technology advances, integrating such tools with other digital health technologies and telemedicine platforms will be essential for enhancing the detection and management of gingivitis and other oral health conditions.

## Conclusion

The mHealth tool GumAI has shown high accuracy in detecting gingivitis and has been well-received by older adults in community settings. While it has not yet reached the level of high-quality tests compared to assessments by a panel of periodontists, its high sensitivity suggests it has potential for community use. Further enhancements are needed to improve specificity. GumAI can potentially address gaps in dental care access, providing a cost-effective and accessible solution for the early detection of gingivitis and oral hygiene management.

## CRediT authorship contribution statement

**Reinhard Chun Wang Chau:** Validation, Formal analysis, Investigation, Resources, Data curation, Writing – original draft, Writing – review & editing, Visualization. **Andrew Chi Chung Cheng:** Conceptualization, Methodology, Software, Validation, Formal analysis, Writing – review & editing. **Kaijing Mao:** Methodology, Software, Validation, Formal analysis. **Khaing Myat Thu:** Methodology, Resources, Data curation, Validation. **Zhaoting Ling:** Resources, Data curation, Validation. **In Meei Tew:** Resources, Data curation. **Tien Hsin Chang:** Data curation, Validation. **Hong Jin Tan:** Data curation, Validation. **Colman McGrath:** Writing – review & editing, Supervision. **Wai-Lun Lo:** Software, Supervision. **Richard Tai-Chiu Hsung:** Conceptualization, Methodology, Software, Validation, Writing – review & editing, Supervision, Project administration, Funding acquisition. **Walter Yu Hang Lam:** Conceptualization, Methodology, Validation, Writing – review & editing, Supervision, Project administration, Funding acquisition.

## Conflict of interests

The authors declare that they have no known competing financial interests or personal relationships that could have appeared to influence the work reported in this paper.
